# Zinc Deficiency in Older Adults Undergoing Rehabilitation With Calcium Pyrophosphate Deposition Disease: A Case Series

**DOI:** 10.7759/cureus.103372

**Published:** 2026-02-10

**Authors:** Naoki Choda, Sayaka Ogura, Kenji Matsumoto, Kazuhisa Domen

**Affiliations:** 1 Department of Rehabilitation Medicine, Shiroyama Hospital, Osaka, JPN; 2 Department of Rehabilitation Medicine, Kansai Rehabilitation Hospital, Osaka, JPN; 3 Department of Rehabilitation Medicine, School of Medicine, Hyogo Medical University, Nishinomiya, JPN

**Keywords:** calcium pyrophosphate deposition disease, hypozincemia, pseudogout, rehabilitation, zinc deficiency

## Abstract

Pseudogout, or calcium pyrophosphate deposition disease (CPPD), is a common form of inflammatory arthritis in older adults. Although metabolic/endocrine factors such as magnesium deficiency and hyperparathyroidism are established risk factors, the role of zinc remains unclear. Since zinc deficiency is prevalent among older adults and zinc influences inflammation control and cartilage metabolism, its potential contribution to CPPD warrants investigation. This study aimed to explore the potential association between zinc deficiency and CPPD in older adults undergoing rehabilitation. We report a case series of five women aged 82-92 years undergoing rehabilitation who developed CPPD. Affected joints included the knees, ankles, wrists, and metatarsophalangeal joints. All patients had serum zinc levels below the reference range (49-67 µg/dL), while other minerals were generally within normal limits. In our study, zinc deficiency was independent of the general malnutrition status. Recurrent CPPD occurred in two patients, particularly those with lower functional status during rehabilitation. This exploratory, hypothesis-generating case series could suggest a potential link between hypozincemia and CPPD in older adults. Lower functional status may also be related to CPPD attacks. While limited by sample size, these findings highlight a clinically relevant hypothesis that warrants further investigation in larger prospective studies.

## Introduction

Calcium pyrophosphate deposition disease (CPPD) is a crystal-induced arthropathy common in older adults [1]. It may present as asymptomatic chondrocalcinosis, acute pseudogout, or chronic CPPD arthritis, resembling rheumatoid arthritis. Acute attacks usually involve mono- or oligoarthritis of the knees and wrists caused by calcium pyrophosphate crystal deposition and subsequent inflammation. Radiographic chondrocalcinosis occurs in up to 50% of the appendicular skeleton in individuals over age 80; however, the proportion of symptomatic cases remains unclear [1].

Several factors contribute to CPPD pathogenesis. Advanced age, prior joint trauma, and osteoarthritis promote cartilage degeneration and crystal deposition [1]. Recent evidence suggests that CPPD is associated with chronic kidney and vascular diseases [1]. Although metabolic/endocrine disorders such as hypomagnesemia, hemochromatosis, and hyperparathyroidism are possible risk factors, they are relatively uncommon in older adults with CPPD [1]. In contrast, available evidence suggests that sex and body mass index are not major risk factors for CPPD, and no specific medications have been consistently identified as established risk factors for CPPD [1].

This study focused on zinc deficiency as a potential risk factor for CPPD. Zinc is an essential trace element involved in many biological processes, and hypozincemia is common in older adults [2]. It regulates inflammation [3] and supports cartilage maintenance [4], suggesting that its deficiency may contribute to CPPD development. In particular, calcium pyrophosphate crystals can trigger acute joint inflammation primarily through activation of the NLRP3 (NACHT, LRR, and PYD domains-containing protein 3) inflammasome, which induces production of the pro-inflammatory cytokine IL-1β (interleukin-1β) [1]. Experimental studies indicate that zinc deficiency can itself activate the NLRP3 inflammasome and enhance IL-1β secretion [3], suggesting that hypozincemia may amplify the inflammatory response to crystal deposition and thereby influence CPPD pathogenesis. Although hypozincemia has been implicated in various disorders [2], its relationship with CPPD has not been previously investigated.

In rehabilitation settings, acute CPPD can cause severe joint pain and reduced mobility [5], while immobility may promote calcium pyrophosphate crystal formation through dehydration [6] or changes in synovial fluid composition [7]. This possible bidirectional relationship between decreased activities of daily living (ADL) and CPPD has not been systematically studied.

This exploratory, hypothesis-generating case series describes five older adults undergoing rehabilitation who developed CPPD. This study aimed to highlight a previously unreported clinical observation-the coexistence of hypozincemia and CPPD in this setting-and to explore its potential association with functional status.

## Case presentation

The diagnosis of CPPD was established either by the identification of calcium pyrophosphate dihydrate crystals in synovial fluid or by fulfillment of the 2023 ACR/EULAR classification criteria, with total scores exceeding 56 points based on clinical, laboratory, and imaging findings [8]. The following case descriptions focus primarily on the clinical course of CPPD episodes during hospitalization. Arthritis episodes were defined clinically by the presence of acute joint pain accompanied by inflammatory signs. Parenthetical day ranges indicate periods during which clinically significant joint pain attributable to CPPD was observed. Day 1 was defined as the first day of admission to the acute care ward.

Case 1

An 85-year-old woman was initially admitted to the acute care ward for subarachnoid hemorrhage and underwent endovascular coil embolization for a cerebral aneurysm on days 1 and 8. During the acute care phase, she developed acute right knee pain (Days 19-22), which resolved without pharmacological treatment. Arthrocentesis of the right knee identified calcium pyrophosphate dihydrate crystals, confirming the diagnosis of CPPD. During rehabilitation, she experienced recurrent episodes of acute inflammatory arthritis affecting the right knee (Days 28-32), right shoulder and neck (Days 45-48), right shoulder again (Days 69-74), and bilateral metatarsophalangeal joints (Days 91-94). Subsequent episodes were supported by imaging findings, including computed tomography (CT) and dual-energy CT [9]. Episodes were managed with oral nonsteroidal anti-inflammatory drugs (NSAIDs), and one shoulder flare required an intra-articular injection of betamethasone sodium phosphate (2 mg). During treatment with loxoprofen (Days 28-37 and Days 45-56; 120-180 mg/day), weight gain was observed; therefore, celecoxib (Days 70-76 and Days 91-133; 100-200 mg/day) was used for the fourth and fifth episodes. Despite recurrent CPPD attacks, her functional status improved with rehabilitation; however, she remained wheelchair-dependent at discharge from the rehabilitation ward. No post-discharge follow-up was conducted at our department.

Case 2

An 86-year-old woman was admitted to the acute care ward after a left middle cerebral artery infarction. During acute hospitalization, she did not experience joint symptoms suggestive of CPPD. She was transferred to the convalescent rehabilitation ward on Day 48. During rehabilitation, she developed recurrent episodes of acute inflammatory arthritis involving the left knee and left hand (Days 50-52, 62-65, 80-83, and 139-142). Radiographic findings of the left knee and hand during the initial episode revealed calcification consistent with CPPD, and arthrocentesis performed during the second episode confirmed the diagnosis. All episodes were treated with oral loxoprofen (Days 50-58, Days 62-74, Days 80-129, Days 139-146, 60-120 mg/day). Her mobility status remained unchanged at discharge from the rehabilitation ward (Day 146). No post-discharge follow-up was conducted at our department.

Case 3

A 92-year-old woman was admitted to the acute care ward (Day 1) for a right femoral neck fracture and underwent total hip arthroplasty on Day 3. After that, she also developed acute cholecystitis requiring subtotal cholecystectomy on Day 26. During the acute care phase, she developed acute inflammatory arthritis of the left hand (Days 30-35), based on self-reported joint pain with swelling and warmth, which resolved without medication. She was transferred to the convalescent rehabilitation ward on Day 40. During rehabilitation, she experienced a recurrent episode of left-hand arthritis (Days 40-43). CPPD was diagnosed based on the clinical presentation and radiographic evidence of chondrocalcinosis in the left wrist. The recurrent episode was treated with loxoprofen (Days 41-54, 120 mg/day). Her functional status improved substantially with rehabilitation, and she was able to ambulate with a walker by discharge from the rehabilitation ward (Day 95). No post-discharge follow-up was conducted at our department.

Case 4

A 90-year-old woman was admitted to the acute care ward because of a right femoral neck fracture. No acute joint symptoms were observed during the early acute care phase. She was transferred to the convalescent rehabilitation ward on Day 36. During rehabilitation, she developed acute inflammatory arthritis (acute pain, swelling and warmth) involving both knees and ankles (Days 40-44). CPPD was diagnosed based on the clinical presentation and radiographic evidence of chondrocalcinosis in the bilateral knees and ankles. Her renal function was impaired (estimated glomerular filtration rate based on serum creatinine, 39-51 mL/min/1.73 m²). Thus, she was treated with acetaminophen (Day 41-53, 500-1200 mg/day) rather than NSAIDs; her inflammatory symptoms improved markedly within one day and had largely resolved by Day 44, although mild pain on movement persisted, and acetaminophen was continued at the patient’s request. She maintained ambulatory function throughout rehabilitation. No post-discharge follow-up was conducted at our department.

Case 5

An 82-year-old woman was admitted to the acute care ward following a left parietal hemorrhage. During the acute hospitalization period, she developed acute left ankle pain (Days 9-13), which was treated with oral loxoprofen (Days 11-16, 180 mg/day). She was transferred to the convalescent rehabilitation ward on Day 28. She experienced recurrent left ankle pain between Days 18 and 35, occasionally accompanied by warmth and swelling; however, symptoms were mild and tolerable without medication. Dual-energy CT was performed to exclude gout, and CPPD was diagnosed based on the clinical presentation and radiographic evidence of chondrocalcinosis in the left ankle. She maintained independent ambulation and was discharged from the rehabilitation ward (Day 86). No post-discharge follow-up was conducted at our department.

Baseline characteristics

Table 1 summarizes the patients’ demographic and clinical characteristics. All patients were older women admitted for rehabilitation following subarachnoid hemorrhage, stroke, or bone fracture. Comorbidities included chronic kidney disease and knee osteoarthritis. The patients had various primary impairments, such as cognitive decline, hemiplegia, hip pain, and generalized weakness, which contribute to their functional limitations and the need for rehabilitation. Body mass index ranged from 19.9 to 25.7 kg/m², and nutritional status was assessed by the Global Leadership Initiative on Malnutrition (GLIM) criteria [10] indicated that one patient had severe malnutrition, while the others were not malnourished.

**Table 1:** Patient demographics * The primary diagnosis was treated surgically. BMI, body mass index; GLIM, Global Leadership Initiative on Malnutrition; SAH, subarachnoid hemorrhage; MCA, middle cerebral artery; CKD, chronic kidney disease (stage based on estimated glomerular filtration rate according to Kidney Disease: Improving Global Outcomes [KDIGO] criteria); DVT, deep vein thrombosis

Case	Age	Sex	Primary diagnosis/comorbidities	Primary impairments	BMI (kg/m²)	GLIM criteria
1	85	Female	SAH* / Postoperative lung cancer	Cognitive decline, generalized weakness	19.9	Not malnourished
2	86	Female	Stroke (Right MCA infarction) / Osteoarthritis of the knees	Cognitive decline, left hemiplegia	20.3	Severe malnutrition
3	92	Female	Right femoral neck fracture* / DVT	Right hip pain, generalized weakness	22.9	Not malnourished
4	90	Female	Right femoral neck fracture* / Osteoarthritis of the knees, CKD (stage 3)	Right hip pain, generalized weakness	25.7	Not malnourished
5	82	Female	Stroke (Left parietal haemorrhage) / CKD (stage 3), Hyperuricemia	Attention deficit, apraxia	20.6	Not malnourished

Overall clinical features and management of CPPD

Affected joints included the knees, shoulders, wrists, ankles, and metatarsophalangeal joints (Table 2). None of the patients had a history of CPPD before admission, but three patients developed episodes during acute-care hospitalization (Cases 1, 3, and 5). Recurrent episodes occurred in two patients during rehabilitation ward admission (Cases 1 and 2). All patients underwent clinical evaluation supported by imaging, including radiography, CT, and dual-energy CT. Synovial fluid analysis in two patients confirmed the presence of calcium pyrophosphate crystals. Most episodes were managed with NSAIDs. For mild symptoms, conservative management, such as local cooling and rest, was used instead of pharmacological treatment. Treatment decisions were made after consultation with the orthopedic department. 

**Table 2:** Affected joints, imaging modality, and treatments of CPPD Days in the “Affected Joints” column indicate the period during which clinically significant joint pain was observed. Days in the “Treatments” column indicate the duration of medication use. Unless otherwise indicated, the patients were treated with oral medication. Day 1 = first day of acute care admission. The days marked with an asterisk refer to the period of acute care admission. CPPD was diagnosed in accordance with the 2023 American College of Rheumatology/European Alliance of Associations for Rheumatology (ACR/EULAR) classification criteria. Cases 1 and 2 were diagnosed by the identification of calcium pyrophosphate dihydrate crystals in the synovial fluid, whereas Cases 3–5 met the criteria based on a total score of >56 points derived from clinical, laboratory, and imaging findings. CPPD, calcium pyrophosphate deposition disease; CT, computed tomography

Case	CPPD episode (day)	Affected joints	Imaging modality	Treatment
1	Day 19*–22*	Right knee	Arthrocentesis	No medications
	Day 28–32	Right knee	Not tested	Loxoprofen (Day 28–37)
	Day 45–48	Right shoulder / neck	CT / CT	Loxoprofen (Day 45–56)
	Day 69–74	Right shoulder	CT	Celecoxib (Day 70–76)+ intra-articular steroid injection (Day 70)
	Day 91–94	Bilateral MTP joints	Dual-energy CT	Celecoxib (Day 91–133)
2	Day 50–52	Left knee / left hand	X-ray / X-ray	Loxoprofen (Day 50–58)
	Day 62–65	Left knee / left hand	Arthrocentesis / Not tested	Loxoprofen (Day 62–74)
	Day 80–83	Left knee / left hand	Not tested	Loxoprofen (Day 80–129)
	Day 139–142	Left knee	Not tested	Loxoprofen (Day 139–146)
3	Day 30*–35*	Left hand	Not tested	No medications
	Day 40–43	Left hand	X-ray	Loxoprofen (Day 41–54)
4	Day 40–44	Bilateral knees and ankles	X-ray / X-ray	Acetaminophen (Day 41–53)
5	Day 9*–13*	Left ankle	Not tested	Loxoprofen (Day 11*–16*)
	Day 18*–35	Left ankle	Dual-energy CT	No medications

Rehabilitation program of CPPD

To our knowledge, no standardized rehabilitation protocols have been established for CPPD. During acute CPPD episodes, rehabilitation programs were temporarily modified according to joint pain and overall clinical condition, with priority given to positioning strategies to minimize excessive joint stress when severe pain was present. As the acute symptoms subsided, therapy initially focused on low-load isometric muscle strengthening, range-of-motion exercises within pain-tolerable limits, and continued attention to joint positioning. The intensity and progression of rehabilitation were adjusted based on pain severity, after which the patients gradually resumed their standard rehabilitation program.

Laboratory findings

Table 3 shows the patient’s blood test results for minerals and trace elements, including zinc. Serum magnesium and calcium levels were mostly normal, except for one slightly elevated magnesium value, probably related to magnesium oxide intake. Iron and ferritin levels varied but showed no consistent pattern of deficiency or overload. Serum copper levels were generally within or slightly above the reference range. However, zinc concentrations were relatively low (47-67 µg/dL) in all patients. Although serum zinc alone may not fully reflect total body zinc status, values below approximately 80 µg/dL generally suggest deficiency [11]. All patients exhibited at least one symptom potentially related to zinc deficiency, such as anemia, anorexia, or perioral dermatitis, but other contributing factors could not be excluded.

**Table 3:** Blood test results of minerals and trace elements Laboratory values represent the first measurements taken during hospitalization when multiple measurements are available. TIBC: total iron-binding capacity. Reference ranges at our institution: Mg, 1.8–2.5 mg/dL; corrected Ca, 8.6–10.1 mg/dL; Fe, 50–170 µg/dL; TIBC, 250–410 µg/dL; ferritin, 5–157 ng/mL; copper, 66–130 µg/dL; zinc, 80–130 µg/dL.

Case	Mg (mg/dL)	Corrected Ca (mg/dL)	Fe (µg/dL)	TIBC (µg/dL)	Ferritin (ng/mL)	Copper (µg/dL)	Zinc (µg/dL)
1	2.2	9.9	51	264	152	123	67
2	2.1	9.1	21	247	141	106	49
3	2.0	9.7	14	151	615	156	59
4	2.7	9.9	63	298	154	132	61
5	2.3	9.3	89	282	214	130	63

Functional outcomes

The Functional Independence Measure (FIM) scores [12] and locomotion status at admission and discharge are summarized in Table 4. Patients 1 and 2, who experienced recurrent CPPD episodes, had relatively low FIM scores on admission and remained wheelchair-dependent throughout hospitalization. Case 3 demonstrated marked improvement, with FIM scores increasing from 55 to 105 points and locomotion improvement from wheelchair use to ambulation with a walker by discharge. Conversely, Patients 4 and 5 had higher baseline FIM scores and maintained good mobility throughout their stay.

**Table 4:** FIM and Locomotion status at admission and discharge The admission and discharge dates corresponded to the stay in the rehabilitation ward. Day 1 = first day of acute care admission. FIM: Functional Independence Measure (total score 126; motor 91, cognitive 35); higher scores indicate greater independence in activities of daily living.

Case	FIM total score at admission (Motor + Cognitive), Day	FIM total score at discharge (Motor + Cognitive), Day	Locomotion status at admission	Locomotion status at discharge
1	36 (24 + 12), Day 32	89 (68 + 21), Day 133	Wheelchair	Wheelchair
2	45 (19 + 26), Day 48	52 (25 + 27), Day 146	Wheelchair	Wheelchair
3	55 (24 + 31), Day 40	105 (78 + 27), Day 95	Wheelchair	Walking with a walker
4	75 (44 + 31), Day 36	102 (71 + 31), Day 65	Walking with a walker	Walking with a walker
5	108 (88 + 20), Day 28	122 (90 + 32), Day 86	Walking	Walkng

## Discussion

We report a case series of five women aged 82-92 years undergoing rehabilitation who developed CPPD. All patients had serum zinc levels below the reference range (49-67 µg/dL), while other minerals showed no consistent pattern and were mostly within normal limits. Those with lower FIM scores and mobility tended to have more frequent attacks. These findings might suggest that hypozincemia and reduced functional status jointly contribute to the development and recurrence of CPPD.

Treatment of CPPD

NSAIDs are commonly used to treat CPPD, although the optimal duration remains uncertain [13]. In older patients, shorter courses are preferred to minimize adverse effects. However, Patients 1 and 2 experienced multiple recurrences, necessitating prolonged NSAID therapy. Notably, Patient 3 achieved symptom control with acetaminophen alone, possibly because she was taking oral magnesium oxide, which has been reported to alleviate pain and inflammation in CPPD [1].

Zinc deficiency and its potential role in CPPD

While previous studies have reported magnesium deficiency, hyperparathyroidism, and hemochromatosis as risk factors for CPPD [1], our patients showed almost normal magnesium and calcium levels and inconsistent iron status. Their underlying conditions were heterogeneous, with no clear unifying risk factors. Conversely, all patients had low serum zinc concentrations, suggesting a possible association with CPPD. Only one patient met the GLIM criteria for malnutrition, indicating that zinc deficiency was not merely a reflection of general nutritional status.

Zinc is important for immune regulation, and its deficiency may heighten inflammatory responses [3]. Mechanistically, zinc deficiency could promote activation of the NLRP3 inflammasome, leading to increased IL‑1β production [3], a pathway also involved in CPPD [1]. Specifically, zinc supplementation has been shown to reduce inflammatory cytokines [14] and C-reactive protein in adults [15]. Additionally, it influences cartilage metabolism via antioxidant activity and structural maintenance [4], while impaired cartilage homeostasis promotes crystal deposition [1]. Thus, zinc may help modulate inflammation and preserve cartilage integrity, which may be relevant to the pathogenesis of CPPD.

Zinc deficiency is common in older adults [16], and CPPD is more frequent in this population [1]. Although confounding factors cannot be excluded, this overlap suggests an association between zinc deficiency and CPPD. However, without comparison to individuals without CPPD, it remains uncertain whether hypozincemia is disease-specific or acts in combination with other factors such as comorbidities (e.g., chronic kidney disease). Functional status might also play a role, as discussed below.

Relationship with functional status

Our findings may suggest a possible interaction between functional status and CPPD, with patients having lower FIM scores experiencing more frequent episodes. Joint pain can reduce mobility [5], while immobility may promote calcium pyrophosphate crystal formation through dehydration [6] or changes in synovial fluid composition [7]. In Case 2, CPPD developed on the paretic side, consistent with previous reports [17] and supporting the idea that immobility may contribute to its onset. Although FIM scores were not assessed during acute hospitalization, patients likely had lower functional status, which may have contributed to the onset of CPPD, even in those with higher FIM scores at rehabilitation admission.

Limitations and perspectives

This study is limited by its small sample size, lack of controls, and observational design. Potential confounding factors, including comorbidities, cannot be excluded. Serum zinc was measured at limited time points and can be affected by factors such as albumin levels, infection, and stress [2]. In addition, the diagnosis of CPPD was heterogeneous, with crystal confirmation in only two cases. However, our findings could support the clinically relevant hypothesis that hypozincemia and reduced ADL are modifiable risk factors for CPPD. Larger studies are needed to clarify this relationship and evaluate the potential benefits of zinc supplementation in preventing CPPD.

## Conclusions

This exploratory, hypothesis-generating case series could suggest a potential association between hypozincemia and CPPD in older adults undergoing rehabilitation. Reduced ADL and mobility may also be related to CPPD attacks, although the causal relationship remains unclear. Despite the small sample size, these findings highlight a clinically relevant hypothesis that should be explored in larger prospective studies.
